# Anti-aflatoxigenic effect of *Lactobacillus rhamnosus* and its synbiotic combination of chitosan/ZnO in milk

**DOI:** 10.1186/s13568-025-01960-z

**Published:** 2025-11-06

**Authors:** Omnia Karem M. Riad, Heba Mohammed Refat M. Selim, Sally T. K. Tohamy, Khaled M. Aboshanab

**Affiliations:** 1 Department of Microbiology and Immunology, Faculty of Pharmacy (Girls), Al-Azhar University, Cairo, 11651 Egypt; 2 Department of Pharmaceutical Sciences, College of Pharmacy, AlMaarefa University, P.O. Box 71666, 11597 Riyadh, Saudi Arabia; 3 Department of Microbiology and Immunology, Faculty of Pharmacy, Ain Shams University, Cairo, 11566 Egypt

**Keywords:** Mycotoxin detoxification, Lactic acid bacteria, Nanomaterials, Probiotics, Microbial synergy

## Abstract

Aflatoxin contamination of milk is a serious health concern. When animals ingested food contaminated with aflatoxin B1 (AFB1), it would be converted into aflatoxin M1 (AFM1) and secreted in the milk. This carcinogenic and hepatotoxic toxin could be overcome by biological methods. Therefore, this study aimed to assess the anti-aflatoxigenic effect of the probiotic *Lactobacillus rhamnosus* (*L. rhamnosus*) ATCC 7469, as well as its synbiotic combination with chitosan ZnO nanocomposite using ELISA. This is carried out by measuring AFM1 concentration in 90 milk samples, including 73 raw and 27 powdered milk samples. The average AFM1 concentration was 11.22 ± 11.31 and 10.62 ± 8.08 µg/kg, which exceeded the international regulatory limits for raw and powdered milk, respectively. All milk samples were treated with *L. rhamnosus* ATCC 7469 and a synbiotic combination of chitosan/ZnO nanocomposite and *L. rhamnosus* ATCC 7469*.* The results showed that the probiotic *L. rhamnosus* ATCC 7469*, *and the synbiotic combination significantly reduced the AFM1 concentration in milk (*p value* ≤ 0.05). The used probiotic bacteria showed binding to AFM1 from 10 to 83.8%, while the binding range increased to 34–92% after treating milk with the synbiotic combination. In conclusion, the biological treatment of milk using the probiotic, *L. rhamnosus* ATCC 7469*, *alone or in combination with chitosan/ZnO nanocomposite, is an efficient method for reducing the AFM1 concentration in milk. This study highlights the use of both metal nanoparticles (as a prebiotic) and probiotics, forming a synbiotic approach to control milk contamination with aflatoxins in the laboratory.

## Introduction

Mycotoxins are fungal metabolites that cause acute toxicity, carcinogenesis, mutagenesis, teratogenicity, immunotoxicity, and estrogenic effects in humans and animals (Awuchi et al. 2022; Khan et al. 2024; Carbonell-Rozas et al. 2025). Mycotoxins are more common in hot and humid climates because they promote mold growth, although they can also be found in temperate zones (Wild and Gong 2009). Mycotoxins, which are commonly found in maize, have been documented to pose a risk to food safety, as they cause acute and chronic consequences such as liver cancer, immunosuppression, inflammation, and respiratory difficulties (James and Zikankuba 2018). Aflatoxin (G1, G2, B1, B2, and M1), fumonisins (B1 and B2), deoxynivalenol, trichothecenes, and ochratoxin A are examples of mycotoxins (Boshra et al. 2023; Olariu et al. 2025). Aflatoxins (AF) are one of the most famous mycotoxins, which are produced specifically by *Aspergillus parasiticus* (*A. parasiticus)* and *A. flavus* (Boshra et al. 2023; Mahjoory et al. 2023).

It is a worldwide concern for human health since it causes hepatocellular carcinoma and contaminates 25% of the world’s crops (Liu and Wu 2010). Contamination of milk with aflatoxin is a major problem, as milk and dairy products are often consumed by humans (Kourti et al. 2025; Jahromi et al. 2025). Aflatoxin in milk products may come from animal feed, which contains AFB1 that is absorbed by the animal’s intestine and then transformed to AFM1 in the liver (Battacone et al. 2003; Olariu et al. 2025; Jahromi et al. 2025). Within 15 min of ingestion of food contaminated with AFB-1, AFM-1 was found in the animal’s blood, and consequently in lactating animals’ milk. Even at sterilization and pasteurization, AFM1 remains stable (Fallah 2010). Hepatotoxicity and carcinogenicity of AFM1 have been documented. The World Health Organization (WHO) reclassified certain substances previously categorized as Group 2 carcinogens to Group 1, based on emerging evidence of their carcinogenicity (WHO 2016).

Investigation of mycotoxins in food is one of the most important examples because of their negative impact on human health (Awuchi et al. 2022; Carbonell-Rozas et al. 2025). Mycotoxins have major health consequences for humans and animals (Edite Bezerra da Rocha et al. 2014; World Health Organization (WHO) 2016). Milk is an important part of the human diet because it provides a natural, high-quality source of bioavailable calcium and proteins, so the quality of milk products has a significant impact on human health at different ages. However, milk can harbor various pathogens and may also be contaminated with other toxins during transfer. Numerous studies have confirmed that milk contains high levels of mycotoxins (Kaan Tekinşen and Cenap Tekinşen 2005; Abdallah et al. 2019; Kourti et al. 2024). AFM1 can be detected in various dairy products from animals that are AFB1-contaminated feedstuffs (Abdallah et al. 2015; Boshra et al. 2024). AFM1 in milk is projected to increase by up to 50% by 2030 due to ongoing contamination trends (Van der Fels-Klerx et al. 2019). The majority of countries have specified the most extreme acceptable level of AFM1 in milk so that consumers everywhere can be confident that purchased milk meets the agreed standards for safety and quality. Economic considerations throughout the world affect regulatory limits, which may vary from one country to another. According to USA regulations and Codex Alimentarius Commission, AFM1 content in milk should not be more than 500 ng/kg (0.5 μg/kg). Moreover, AFM1 in milk is limited to a maximum of 50 ng/kg (0.05 μg/kg) by the European Community (World Health Organization (WHO) 2016).

Various chemical, physical, and biological approaches have been tested for their capacity to bind with aflatoxins in feed and food products to remove AF from feed and food (Taheur et al. 2017; Kourti et al. 2025; Jahromi et al. 2025; Hou 2025). Probiotics, which are live microorganisms that provide advantages for both human health and the food business, are one type of biological approach that is employed. One of the many probiotics frequently used in the dairy products sector is *Lactobacillus rhamnosus* (Balthazar et al. 2018; Silva et al. 2018; Sperry et al. 2018)*.* Probiotics can treat or prevent several diseases such as diarrhea, allergies, and other ailments (Martins et al. 2005; Sperry et al. 2018). Probiotics inhibit mold growth and, as a result, the generation of AF in meals (Boshra et al. 2023, 2024). Furthermore, several strains have been observed to complex with aflatoxin effectively in contaminated milk and liquid culture media. *L. rhamnosus* GG is the most researched probiotic microorganism for mycotoxin binding (Assaf et al. 2018a). In terms of the binding mechanism between LAB and AF, it was thought that AF binds to polysaccharides and peptidoglycans found in the cell walls of lactic acid bacteria. In previous research, this binding has been found to incorporate Van der Waals and hydrogen bond interactions as well (Shetty and Jespersen 2006; Kim et al. 2017). Also, it was elucidated that the ability of LAB such as *L. rhamnosus* GG to make biofilm on different types of surfaces under specific conditions could be utilized in the decontamination of AFM1-contaminated milk by their binding to biofilm (Assaf et al. 2019a).

Prebiotic use has been encouraged in recent years to enhance the actions of the probiotic bacteria. Dietary fibers are good examples of prebiotics, including dietary oligosaccharides, such as lacto-sucrose, galactooligosaccharides (GOS), fructooligosaccharides (FOS), and isomalto-oligosaccharides (Awuchi et al. 2022; Khan et al. 2024). A recent study confirmed that chitosan in high concentration has a prebiotic-like effect and enhances the actions of probiotic bacteria (Zhang et al. 2020). Toxigenic fungi could be controlled by some nanoformulations (Bocate et al. 2019), such as copper-chitosan nanocomposite-based hydrogels, which are effective in suppressing the growth of various toxigenic fungi (Abd-Elsalam et al. 2020). Also, silver nanoparticles were found to inhibit *A. flavus* growth and aflatoxin production to various extents (Al-Othman et al. 2014). The purpose of this study was to test the anti-aflatoxigenic ability of *L. rhamnosus* ATCC 7469 in milk. In this study, we present a novel synbiotic formulation combining *L. rhamnosus* ATCC 7469 with a chitosan/ZnO nanocomposite to increase the anti-aflatoxigenic effect*,* taking advantage of the prebiotic effect of chitosan and the fungicidal effect of nanoformulation. This work aimed to evaluate the anti-aflatoxigenic potential of this specific combination in a dairy matrix, offering a dual-action strategy that leverages both biological and physicochemical mechanisms. Our findings contribute a new perspective to food safety interventions by integrating microbial functionality with nanotechnology-based enhancement.

## Materials and methods

### Bacterial strain and culture conditions

*L. rhamnosus* ATCC 7469 was purchased from MIRCEN, Faculty of Agriculture, Ain Shams University, Cairo, Egypt. It was supplied as an active culture. In broth of de Man Rogosa Sharpe (MRS) (Oxoid, England)*, L. rhamnosus* ATCC 7469 was cultured at 37 °C for 24 h under aerobic conditions. It was stored and kept in Luria–Bertani (LB) broth containing glycerol (50:50) at − 20 °C for long-term preservation (Miller 1972).

### Collection of milk samples

Samples of milk were randomly taken from Egyptian markets from various governorates in the period from September 2022 to June 2023. A total of 90 milk samples were obtained, including powdered milk (n = 17) and raw milk (n = 73). Samples were collected in sterile containers, stored frozen at − 20 °C until they were tested and processed. AFM1 was quantified in the whole collected milk samples using an ELISA kit (Cat. no. E4566, Biovision, CA, USA). About 27 samples of the higher limits of AFM1 were subjected to further treatment by *L. rhamnosus* ATCC 7469 and a combination of *L. rhamnosus* ATCC 7469 and chitosan/ZnO nanocomposite, which was previously prepared and characterized (Badawy et al. 2020)**.**

### Pretreatment of milk samples

To ensure accurate extraction and quantification of AFM1, the sample undergoes pretreatment steps such as centrifugation and filtration to eliminate fats and impurities that may interfere with the process. Extraction was done using acetonitrile (HPLC grade, Fine-Chem Ltd., India) as an organic solvent, as previously reported (Vaz et al. 2020)**.** Pretreatment steps were done as recommended by the manufacturer’s instructions. For the raw milk samples, approximately 4 mL of acetonitrile was added to a 50-mL centrifuge tube containing 1 mL of milk sample, and the mixture was mixed for 5 min. This was followed by centrifugation at 4000 rpm at 25 °C for 10 min. Then, a total of 2.5 mL of supernatant was dried in a water bath, followed by the addition of 1 mL of the redissolving solution from the ELISA kit. Finally, about 50 μL was taken for determining the AFM1 concentration using ELISA analysis as previously reported (Vaz et al. 2020).

### Pretreatment of powdered milk samples

After adding 20 mL of sample extract solution (supplied with the ELISA kit) to a 50-mL centrifuge tube containing five grams of milk powder, oscillation for 5 min was done, followed by centrifugation at 4000 rpm at room temperature for 10 min. A total of 2.5 ml of supernatant was dried in a water bath, followed by adding 750 μL of the redissolving solution (present in the ELISA kit). Then, 50 μL was taken for measuring AFM1 using ELISA analysis as previously reported (Kourti et al. 2024).

#### Aflatoxin AFM1 quantification in milk

The milk samples were quantified for AFM1 before and after the addition of *L. rhamnosus* ATCC 7469 and the synbiotic combination.

#### Quantification of AFM1 in milk samples before any further addition

A total of 50 μL of the pretreated milk samples was taken for quantification by the ELISA kit (Biovision Cat. No. E4566) as per the manufacturer’s instructions. Absorbance was read at a wavelength of 450 nm with a microplate reader. Concentrations were calculated as recommended by the manufacturer (Kourti et al. 2024).

#### Quantification of AFM1 in milk after addition of L. rhamnosus ATCC 7469

About 10 μL of 1 McFarland *L. rhamnosus* ATCC 7469 suspension (corresponding to 3 × 10^8^ CFU/mL), prepared in saline, was incubated with 1 mL of milk sample at 37 °C for 24 h (Boshra et al. 2023). Then, quantification of AFM1 was done by ELISA as previously described (Vaz et al. 2020; Kourti et al. 2024).

#### Quantification of AFM1 in milk after synbiotic combination (L. rhamnosus ATCC 7469 and chitosan/ZnO nanocomposite)

A total of 100 μL of chitosan/ZnO nanocomposite (9.5 × 10^–4^ mg/mL) was added to 1 mL of milk samples along with 10 μL of 1 McFarland *L. rhamnosus* ATCC 7469 suspension (corresponding to 3 × 10^8^ CFU/mL) and incubated at 37 °C for 24 h (Boshra et al. 2023). ELISA quantified AFM1 in both raw and powdered milk samples (Boshra et al. 2024; Kourti et al. 2024).

### Statistical methods

To discover significant differences between the three study groups, SPSS version 23.0 (SPSS Inc., Chicago, IL, USA) was utilized. Median and range were employed to describe the quantitative data. The Kruskal–Wallis test was used for more than two study group comparisons in case of *non-normally distributed* data.

## Results

### Aflatoxin M1 concentration in raw milk and powdered milk

In this study, ninety samples (n = 90) of powdered and raw milk were taken from various local marketplaces and retail stores in different Egyptian governorates and then analyzed for contamination with AFM1. The ELISA technique used to quantify AFM1 in milk revealed that the mean concentration of AFM1 in all collected samples was 11.10 ± 10.74 ng/L (mean ± SD), with a standard error of 1.24 ng/L*.* By comparing detected AFM1 concentrations to different accepted limits, it was found that all samples, whether raw milk or powdered milk, are exceeding the Egyptian, U.S. Food and Drug Administration, Codex Alimentarius Commission, and European Commission regulations. The results confirmed that the concentrations in both raw and powdered milk samples were statistically higher than the accepted limits (*p* < 0.05). As shown in Table 1**,** there is a wide range of AFM1 concentrations. The median AFM1 concentration in samples collected from powdered milk was higher than in samples collected from raw milk, although there was no significant difference between them (*p value* = 0.546).

**Table 1:** Aflatoxin M1 concentration in raw milk and powdered milk

Sample type	AFM1 concentration (µg/kg)			
	Mean ± SD	Median	Range	*P* value
Powdered milk (N = 17)	10.62 ± 8.08	10.2	0.8–28.60	0.546
Raw milk (N = 73)	11.22 ± 11.31	6.5	0.70–47.00	
Total (N = 90)	11.10 ± 10.74	7.70	0.7–47.00	

### Anti-aflatoxigenic effect of L. rhamnosus ATCC 7469

From ninety milk samples, 27 samples with the highest level of AFM1 concentration were selected for further investigations on the effect of *L. rhamnosus* ATCC 7469*.* Table 2 shows that after the addition of *L. rhamnosus* ATCC 7469*,* the mean concentration values of AFM1 in milk samples were significantly (*P* < 0.05) lower than milk samples without any additives, which suggests that *L. rhamnosus* ATCC 7469 has a strong effect in decreasing AFM1 in milk. The range of percentages of AFM1 binding by *L. rhamnosus* ATCC 7469 was 10% to 83.8%.

**Table 2:** AFM1 concentration in milk samples under different treatments

Groups	AFM1 Concentration (µg/kg)			
	Mean ± SD	Median	Range	Interquartile range (IQ)
A (N = 27) Control milk samples (original)	22.88 ± 10.78	23	9.30–47.00	12.6–31
B (N = 27) After the addition of *L. rhamnosus* ATCC 7469	11.38 ± 7.84	9	2.10–29.00	4.8–16
C (N = 27) After the addition of *L. rhamnosus* ATCC 7469 and Chitosan/ZnO	6.69 ± 5.03	6.1	1.20–19.00	2.1–9.6

Group A and B: *p value* < 0.001*; Group A and C: *p value* < 0.001*; Group B and C: *p value* < 0.091

### Anti-aflatoxigenic effect of the synbiotic combination

The effect of the synbiotic combination of Chitosan/ZnO nanocomposite and *L. rhamnosus* ATCC 7469 was detected by the addition of this combination to the same samples (n = 27) with the highest level of AFM1 concentration. Table 2 represents that after the addition of the synbiotic combination of* L. rhamnosus* ATCC 7469 and Chitosan/ZnO nanocomposite*,* the mean concentration values of AFM1 in milk samples were significantly lower than milk samples without any additives, which suggests that the combination of* L. rhamnosus* ATCC 7469 and Chitosan/ZnO nanocomposite has a significant effect (*P* < 0.05) in decreasing AFM1 in milk. The range of percentages of AFM1 binding by the synbiotic combination increased from 10–83.8% to 34–92%.

### The anti-aflatoxigenic effect of L. rhamnosus ATCC 7469 before and after the addition of chitosan/ZnO nanocomposite

**Fig. 1:**
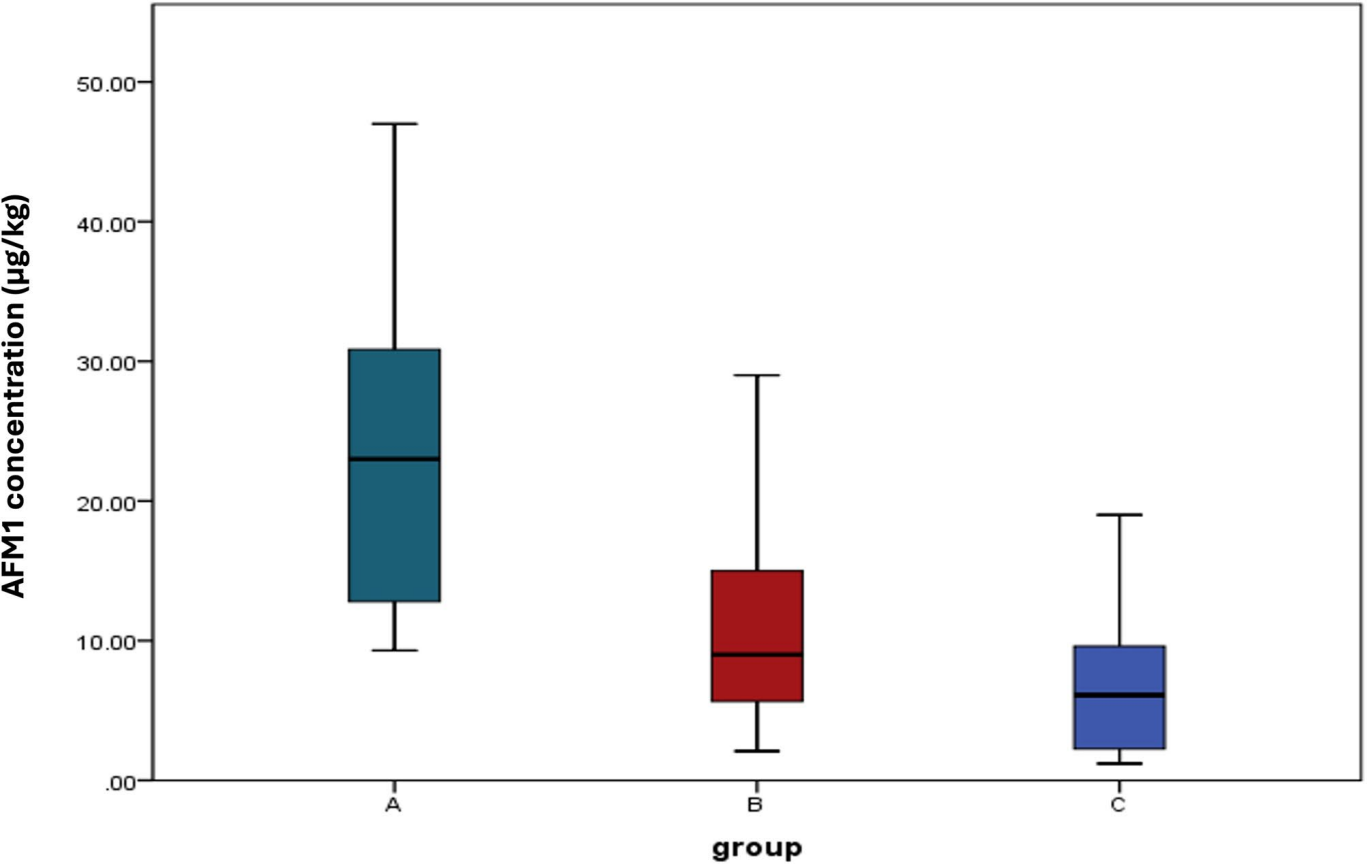
Box and whisker plot showing aflatoxin amount distribution between the three studied groups (A, B and C)

Although *L. rhamnosus* ATCC 7469 in both cases has a remarkable anti-aflatoxigenic effect, our results revealed that the aflatoxin amount in group C (n = 27) & group B (n = 27) has no significant difference between them (*P* > 0.05) as displayed in Table 2**.** Moreover, the Box and whisker plot showing aflatoxin amount distribution between three studied groups, A, B, and C, revealed significant differences between groups A and B (*P* < 0.001) and A and C (*P* < 0.001), while no significant (*P* > 0.05) results were observed between groups B and C **(**Fig. 1).

## Discussion

This study aimed to evaluate the anti-aflatoxigenic properties of *L. rhamnosus* ATCC 7469 in milk alone and in combination of chitosan/ZnO nanocomposite. Utilizing the fungicidal action of the nanoformulation and the prebiotic impact of chitosan, the goal is also to explore whether the chitosan/ZnO nanocomposite may enhance the anti-aflatoxigenic effect of *L. rhamnosus* ATCC 7469. The Egyptian Ministry of Health mandated that raw milk and dairy products be AFM1-free. In different countries, the incidence of AFM1 in milk has been confirmed by researchers who reported high levels of AFM1 (Aiad and Sobhy 2013). In this study, results documented AFM1 in 100% of analyzed raw milk and powdered milk samples with a level ranging from 0.70 to 47.00 μg/kg. All positive samples are above the regulations of Egypt, the European Commission, the U.S. Food and Drug Administration, and the Codex Alimentarius Commission regulations (World Health Organization (WHO) 2016). The findings of this study were in good agreement with those of several other investigations (Giovati et al. 2015; Bukari et al. 2020). Another study in Egypt stated that AFM was detected in every sample tested, and 14 samples (70%) showed concentrations exceeding the acceptable level in the European Commission (0.05 μg/kg). Their AFM levels were from 0.02 to 0.19 μg/kg (Abdallah et al. 2019). These findings necessitate the close monitoring of the animal feed in Egypt, and the application of Good Agricultural Practices (GAP) could restrict AF contamination in animal feed; however, this cannot guarantee that it will not be found again (Karlovsky et al. 2016).

In another study, AFM1 was present in every examined sample of raw milk collected in Pakistan (Iqbal and Asi 2013). Elzupir et al*.* found that 95.5% of milk samples collected in Sudan contain a high concentration of AFM1 (Elzupir et al. 2012). On the other hand, the percentage recorded in this study was greater than that documented in Libya by Elgerbi et al. who recorded that a high percentage of collected raw milk samples were positive (71%) with variable levels of AFM1 concentrations from 0.03 to 3.13 μg/L (Elgerbi et al. 2004). Also, these results were higher than those detected by Tekinşen and Eken (2008) in Turkey, who revealed that among the collected milk samples, AFM1 with levels ranging from 0.010 to 0.630 μg/L was detected in 67% of samples (Tekinşen and Eken 2008). A study by Awasthi et al. (2012) on unpasteurized and pasteurized milk samples detected AFM1 at a concentration ranging from 0.056 to 0.082 ppb in both types of analyzed milk samples, reflecting that the concentration range of AFM1 detected in milk is not affected by the pasteurization process (Awasthi et al. 2012).

This study’s conclusions are broadly in line with those of previous Egyptian investigations and point to a severe public health issue for the Egyptian people as they are exposed to AFM1 concentrations in milk that are higher than the accepted international limit. Furthermore, the total daily intake of mycotoxins from various types of food may be an important risk factor to consider. Thus, it is essential to create more effective milk detoxification processes. The application of Good Agricultural Practices (GAP) could restrict AF contamination in animal feed, but it cannot guarantee that it will not be found again (Karlovsky et al. 2016). Thermal procedures, pasteurization, autoclaving, and different food processing procedures do not affect AFM1; thus, other procedures are tested to decontaminate milk (Park 2002). New emerging techniques include using probiotic bacteria, chitin, and treated crustacean shells, which have been recently studied in several studies (Assaf et al. 2018a, b, 2019a, b).

Probiotic bacteria, e.g., *Lactobacillus* spp., have several biological activities, including antibacterial, anticancer, and antitoxin activities (Salem-Bekhit et al. 2023; Abu-Elfotuh et al. 2023; Zaghloul et al. 2023; Riad 2024; Riad et al. 2025). Probiotic yeasts and lactic acid bacteria have been used to decontaminate milk and liquid foods from AFM1 for several years due to their safety, as well as their great potential to attach to mycotoxins as adsorbents (Assaf et al. 2019b). Different lactic acid bacteria, such as *L. fermentum, L. plantarum,* and* L. rhamnosus* ATCC 7469, are commonly utilized nowadays for fermentation, especially milk fermentation (Santiago-López et al. 2018). Thus, it is strongly recommended to use microbial binders like those found in dairy products (Assaf et al. 2019b). Serrano-Nino et al*.* revealed that strains of probiotics such as *L. acidophilus* and *L. plantarum* bind to AFM1, leading to a remarkable decrease in the bio accessible level of AFM1 (Serrano-Niño et al. 2013).

Therefore, mycotoxins in food can be eliminated or removed by LAB through physical attachment or bio-transforming mechanisms (Hamad et al. 2025). Various studies have established the effectiveness of lactic acid bacteria’s adsorption in removing AFs from liquid solutions (Liew et al. 2018; Salem-Bekhit et al. 2023). According to a different study, some probiotics can adsorb AFs in dough (cultured dairy product) while it is fermenting and being stored. However, the type of probiotic determines the adsorption rate of AFM1 as previously reported (Essia Ngang et al. 2015; Hamad et al. 2025).

This study investigated the effect of 3 × 10^8^ CFU/mL of *L. rhamnosus* ATCC 7469 on aflatoxin concentration in selected samples of high aflatoxin content. Results revealed that after the addition of *L. rhamnosus* ATCC 7469 to milk samples*,* AFM1 mean concentration values were significantly lower than milk samples without any additives, and the range of percentages of AFM1 binding by *L. rhamnosus* ATCC 7469 was 10–83.8%. This suggests that *L. rhamnosus* ATCC 7469 has a strong effect in decreasing AFM1 in milk. This result is consistent with Liew and his colleagues, as they confirmed that the concentration of 10^9^ live cells of* L. casei* showed a high binding capacity of AFB1, which reached 98%. Another similar result recorded that 2 × 10^10^ CFU/mL of *L. acidophilus* and *Bifidobacterium longum* lower the AFB1 level to less than 0.05 (Apás et al. 2014).

Moreover, Anfossi et al. studied the anti-aflatoxigenic effect of different strains of *Lactobacillus *spp. and *Bifidobacterium *spp (Anfossi et al. 2010)*.* They concluded that 25.94% of AFM1 was successfully removed regardless of contact hours. Similarly, a study tested different procedures to measure the degree of binding of AFM1 in milk with *L. rhamnosus* (Assaf et al. 2018a). They concluded that using *L. rhamnosus* could result in the binding of approximately 63% of AFM1, and hence it could be used as a milk additive to strongly detoxify milk. In a recent study, the ideal *L. rhamnosus* to *S. cerevisiae* ratio, incubation duration, and temperature for effective AFM1 detoxification from milk were determined using a Box–Behnken design. Contaminated milk samples showed up to 98.4% AFM1 detoxification. These encouraging findings offer a low-cost, low-time, and safe way to eliminate the problem of milk contamination with AFM1 (Salem-Bekhit et al. 2023).

In the current study, the addition of the synbiotic combination composed of the tested probiotic bacteria, *L. rhamnosus* ATCC 7469*,* and chitosan/ZnO nanocomposite was carried out, exploiting the advantages of adding chitosan with the assumed anti-aflatoxigenic and prebiotic effect, in the form of ZnO nanocomposite to enhance the action of chitosan, in addition to its anti-aflatoxigenic effect. Limited studies have been carried out to evaluate the synbiotic combination of probiotic bacteria and chitosan metal oxides. Documents indicated that chitosan ZnO nanocomposites are stronger than ZnO nanocomposites in killing pathogenic fungi such as *C. albicans* (Dananjaya et al. 2018).

Moreover, it was concluded that copper-chitosan nanocomposite has antifungal activity against *A. flavus* not only by damaging the fungal cell wall but also by downregulating the genes involved in aflatoxin biosynthesis (Abd-Elsalam et al. 2020). Another study revealed that the encapsulation of chitosan nanoparticles enhanced *L. plantarum* RM1’s preventive effectiveness against AFM1-induced hepatorenal toxicity in rats (Hassanen et al. 2023).

Results of the present study indicated that a concentration of 9.5 × 10^–4^ mg/mL chitosan/ZnO nanocomposite added to 3 × 10^8^ CFU/mL of *L. rhamnosus* revealed that AFM1 mean concentration values in milk samples after the addition of a combination of* L. rhamnosus* ATCC 7469 and chitosan/ZnO nanocomposite were significantly lower than milk samples without any additives, which suggests that the combination of* L. rhamnosus* ATCC 7469 and chitosan/ZnO nanocomposite has a strong effect to decrease AFM1 in milk. This study compared the levels of AFM1 in the milk samples treated with *L. rhamnosus* ATCC 7469 alone to those treated with a combination of *L. rhamnosus* ATCC 7469 with chitosan/ZnO nanocomposite. The results showed no significant difference between the two treatments. However, the range of AFB1 binding percentages was higher with the combination of *L. rhamnosus* ATCC 7469 and chitosan/ZnO nanocomposite (34–92%) than with the addition of *L. rhamnosus* ATCC 7469 only (10–83.8%). This suggests that the chitosan/ZnO nanocomposite has a probable prebiotic effect on *L. rhamnosus,* ATCC 7469, which could be further investigated to reach a significant level. One limitation of this study was the lack of testing the effect of using chitosan/ZnO nanocomposite alone as a control group. However, this control group will be considered in our future work. Moreover, in vivo studies are recommended to evaluate similar synbiotic combinations in reducing AFM1 in milk.

As a conclusion, this investigation revealed the prevalence and degree of AFM1 contamination in Egyptian milk. AFM1 concentrations in the most examined milk samples were higher than the international allowed limit, implying that drinking dairy milk in Egypt poses a serious public health risk. To develop a mycotoxin incidence database, continuous and permanent monitoring is strongly suggested. Food control agencies should apply stricter regulations to control mycotoxin levels in milk. Finding safe and effective creative techniques to minimize aflatoxin levels in milk is critical. This study sheds light on the use of both metal nanoparticles and probiotics in combination to control milk contamination with aflatoxins in the laboratory. Results revealed that *L. rhamnosus* ATCC 7469 has a strong anti-aflatoxigenic effect. However, the synbiotic combination of Chitosan/ZnO nanocomposite and *L. rhamnosus* ATCC 7469 requires further studies to maximize their anti-aflatoxin effect and reach the highest level of performance for further use in the field of milk production, which will significantly be reflected in improving milk quality.

## Data Availability

All data generated or analyzed during this study are included in this published article.
